# Supplementary material for: Assessment of end-of-life vehicle recycling: Remanufacturing waste sheet steel into mesh sheet
Source: PLoS One. 2021 Dec 7;16(12):e0261079. doi: 10.1371/journal.pone.0261079 (PMC8651122; doi:10.1371/journal.pone.0261079)
Supplement: Attachment — Submitted filename: Rebuttal letter PONE-D-21-27091.docx [file pone.0261079.s001.docx]

Rebuttal Letter for Manuscript PONE-D-21-27091" Assessment of End-of-life Vehicle Recycling: Remanufacturing Waste Sheet Steel into Mesh Sheet"

The responses to the reviewers’ comments appear here in red text. The changes in the revised manuscript also appear in red text.

**Reviewer #1**: 1. The manuscript assessed producing Mesh Sheet from recycled waste sheet steel.

2. The manuscript’s strength is based on data in the manufacturing plant in Iraq. The weakness is that it is unclear whether these findings can be applied to other regions since the assessment results are based on a specific manufacturing plant.

I apologize that this point was not clear in the original manuscript. The study is not based on a single physical plant, but on a model plant developed based on literature data worldwide. I have added a paragraph in the revised manuscript clarifying this point (page 5, lines 3-14).

3. Provide a point-by-point list of my major recommendations for the improvement of the manuscript;

In Conclusion, include any quantitative findings from the evaluation.

The evaluation results should be graphed in addition to tables to facilitate visual understanding.

The table format was chosen as there was a lot of data to include in the paper, and much of it is not appropriate for graphing (no obvious dependent and independent variables). In addition, the journal does not accept repetition of data in both graph and table form. However, if the reviewer can suggest some specific tables that they feel would be more informative in graph form, I will be happy to make this change.

4. If necessary, provide a point-by-point list of my minor for the improvement of the manuscript.

On page 11, Line 4, the reference source is not found.

On page 12, Line 6, the reference source is not found.

On page 14, Line 3, the reference source is not found.

On page 15, Line 5, the reference source is not found.

On page 17, Line 12, the reference source is not found.

On page 19, Line 22, the reference source is not found.

On page 20, Line 15, the reference source is not found.

On page 21, Line 6, the reference source is not found.

On page 23, Line 9, the reference source is not found.

These link errors have been corrected.

On page 15, Line 44, what is the definition of ‘d’?

Here, the unit “/d” is per day. As “d” is the recommended SI unit/abbreviation for “day,” it has been used in this manuscript.

On page 29, Line 11, MSS from MSS is not correct.

Thank you for bringing this to my attention. I have made the change “MSS from **WSS**”.

**Reviewer #2:** 1. Proper management of ELVs, including remanufacturing, recycling and reuse, is a necessary task towards sustainable development. It is worth making continuous efforts to enhance ELV management practices. The study presented in this manuscript is encouraged. It is hoped that the manuscript can be improved with the following suggestions.

Thank you for the kind feedback. I hope that the following changes to the manuscript now make it suitable for publication.

2. Many missing links exist throughout the manuscript which are shown as “Error! Reference not found.” and must be corrected. These references, based on the context, may provide fundamental information for this study. Unable to refer to those references, the discussion developed in Method and Results sections seems to be interrupted.

These link errors have been corrected. These were references to the Figures and Tables. Apologies if this made it more difficult to review my manuscript.

3. (Epsilon) ecc, (Epsilon) rem and (Epsilon) rec are not introduced in the text. Thus, the rationale for Eq.(12) to (14) can not be understood.

Epsilon refers to the eco-cost saving. These symbols were indeed defined in the paragraph preceding Eq 12-14. Therefore, no change has been made in response to this comment.

4. Weights of engine and transmission consist of a large proportion of total weight of automobiles. It seems that the author counts their weights in estimating economical potential; however, only exterior components are considered in the feasibility calculation in the study. How are they considered in WSS? Is it practical to assume 80 m^2^ of sheet can be recovered by ~698 kg per car for remanufacturing as stated in the Results section?

The available WSS was calculated assuming that an average of 80 m^2^ can be recovered from each car to prevent manufacturing of new structural sheet steel of (~698 kg).

5. Table 1 entitles “Weights of the components of WEPrD”; however, Table 1 contains weights for pre-disassembly, post-disassembly and expanding. The title of Table 1 may need to be revised.

I have revised the title of Table 1 to read “Calculated weights of the individual components used to calculate W_EPrD_, W_EPoD_, and W_EEx_” to clarify this.

Besides, it is suggested to indicate the nature of values by notations, such as “estimated”, “calculated” and etc, if data presented in the table are generated in this study, to clarify whether those values are retrieved from literature or calculated results of this study.

Thank you for this suggestion; this has been revised throughout the paper.

6. Opinions from the author on differences among Reuse, recycling and remanufacturing of WSS may be explained more explicitly, regarding technical, economical and environmental feasibility. It is suggested to revise the discussion to illustrate the comparison among these processes from the viewpoint of the author.

Some further discussion has been added on this point (page 13, line 16-22).

7. There are some items in Results for discussion which were not mentioned in previous Introduction and Method sections, such as, remanufacturing feasibility assessment, and etc. It is suggested to explain their relationships with other indexes.

The section titled “Remanufacturing feasibility assessment” discusses the results of the methods described in the section “Calculation of feasibility indexes”. I have added a sentence at the start of the former to explain this.

8. Quite a few paragraphs are used to discuss ASR and other materials in the disassembly plants. It is suggested to focus discussions on how WSS may contribute to MSS with technological or management strategies.

ASR is a current problem in the field, and its volume needs to be greatly reduced. Therefore, it is important to show the feasibility of remanufacturing WSS into MSS to increase the efficiency of dismantling, recycling, smelting, and landfill strategies by reducing ASR.

WSS is the raw material to remanufacture MSS, and since it is a solid waste management strategy, its sustainable development should include the dimensions of technical, economic, social, environmental, and management, which interact and interconnect to close the loop globally among the car industry and metal forming industry. This is important because many countries do not manufacture cars and cannot extend the Original Equipment Manufacturer responsibility to cope with end-of-life cars.

9. The manuscript is suggested to provide page numbers for both reviewing and reading purposes.

Page and line numbers have been added to aid reading and reviewing.

10. Most terms have full names followed by abbreviations when they first appear in the manuscript; however, there are still some missing. Also, some typos exist in the text. Please double check for accuracy.

The entire paper has been revised again considering this advice.
