# Supplementary material for: Assessment of end-of-life vehicle recycling: Remanufacturing waste sheet steel into mesh sheet
Source: PLoS One. 2021 Dec 7;16(12):e0261079. doi: 10.1371/journal.pone.0261079 (PMC8651122; doi:10.1371/journal.pone.0261079)
Supplement: Attachment — Submitted filename: PONE-D-21-27091R1 response to reviewers.docx [file pone.0261079.s002.docx]

Responses to the reviewer for Manuscript PONE-D-21-27091R1 "Assessment of End-of-life Vehicle Recycling: Remanufacturing Waste Sheet Steel into Mesh Sheet"

The responses to the reviewers’ comments appear here in red text. The changes in the revised manuscript also appear in red text.

**Reviewer #1:** This decision was based on the following two points.

The table showing the results is merely a list of numbers, without any quantitative or qualitative analysis. There is a lot of value in organizing the results into graphs instead of tables, but the author does not do so.

There are many tables listing the results, and it is a little unclear which one the reviewer is referring to. As many of the results have ranges of values, which are difficult to express in figure format, I chose the use of tables. In my previous response, I requested that the reviewer clarify which specific tables they would like to see in figure format to make this change. Without this information, it is difficult to comply with this request. As noted before, much of the data is not appropriate for graphing (no obvious dependent and independent variables), so graphing would make the data less easy to understand.

I provide a summary of the tables and the reasons why most of them are better presented as tables. The only viable data for presentation in figure form (in my opinion) appears in Tables 8 and 12. Hence, I have plotted these data as figures in an attempt to address the reviewer’s concern.

Table 1: defines symbols and gives weight values used in calculations. These values are not results, and have no dependent variable.

Tables 2: it would be difficult to label the regions on a figure, and three different data sets are shown in one figure. It would be an inefficient use of space to plot these data. In addition, the calculated ranges would be difficult to express in a figure.

Table 3: as there is no common dependent variable, the comparison of the reuse and remanufacturing efficiencies is thought to be easier in table format.

Table 8: This table could be plotted as a figure. I have made this change (new Figure 3, as shown below).

Tables 4, 5, 6, 7, 9, 10, 11: No common dependent variable, many datasets in one table, inefficient to plot, ranges difficult to express in figures.

Table 12: These data could be presented as a figure. I have made this change (new Figure 4, as shown below).

The conclusion lacks the results of quantitative or qualitative analysis.

I respectfully disagree with the reviewers comment, and apologize if the analysis was unclear. Without specific comments, it is difficult to address this concern. The main conclusion of the paper is related to the overall sustainability of remanufacturing waste sheet steel into mesh sheet steel. A detailed quantitative analysis was provided in the paper, which was condensed into an overall sustainability index as a final solution. This value is considered a quantitative conclusion. This simple way of comparing the various proposed remanufacturing scenarios provides a method for policy makers and ELV processing plants to evaluate the feasibility of the process. The paper provides a clear conclusion regarding the feasibility of the proposed process and its implications and potential benefits in the ELV processing field and automotive industry as a qualitative conclusion.

**Reviewer #2**: 1. Figure 1 and 2 are attached after the text should be provided with their Titles? According to the context, is Figure 1 supposed to show a remanufacturing process for converting WSS from ELVs into MSS?

Following the formatting requirements of the journal, the figure captions were placed in the main text. You can find them on page 6 (lines 6-7) and page 7 (line 2).

2. In equation (14), (epsilon) rec and rem are functions of ecc and CO2, which are defined in equation(12) and (13) and they are explained in the paragraph. However, (epsilon) rec and rem must have different values of ecc and CO2 since their Mvss, Mco2, and vss, right? My question is how are those values determined? They should be also be stated in this Methods section.

Apologies if this was not clear in the original text. The terms ε_rem_ and ε_rec_ were calculated using Eq. (12) where ε_c_ is substituted by ε_rem_ or ε_rec_ as relevant. The reviewer is correct that M_VSS_ is calculated using different values for each case, but ε_VSS_ and M_CO2_ are the same for both recycling and remanufacturing as they are based simply on the savings related to 1 kg of steel. The ε_c_ of VSS is 0.55 USD/kg and the *M_CO2_* is 1.559 kgco_2_/kg_VSS_.

In the case of recycling 1 m^2^ of recovered WSS (assuming 100% recycling), the production of 7.8 kg of VSS, giving an M_VSS_ value of 62.4 kg (assuming that the WSS is used for mesh-steel applications).

In the case of remanufacturing, 1 m^2^ of MSS can prevent 7.8 kg of VSS being produced, with upper and lower bounds of M_VSS_ of 62.4 kg and 698 kg, respectively. The upper bound assumes that the steel is used for sheet-metal product applications and that the eco-design standards are satisfied.

Based on the number of end-of-life vehicles given in the scientific literature, the corresponding data for the recovered and remanufactured WSS was used to calculate the lower and upper bounds of economic feasibility in the form of eco-cost saving and CO_2_ prevention.

I have added a discussion of this in the revised manuscript (page 11, lines 8-14).

3. The estimation of 754 kg steel/ELV recycled from ~ 1 t/ELV, p10, includes weights of engine and transmission which are not considered as WSS. It should be distinguished clearly by using weights of vehicles with reuse, remanufacturing and recycling alternatives in discussion of the economical and environmental efficiencies in the manuscript.

The value of 754 kg/ELV is the total amount of recyclable waste steel that can be recovered from an ELV (with a total weight of typically ~ 1 t). Further, 62.4 kg of the total 754 kg is the net weight of WSS which can be recovered, and its surface area can be amplified by 11.5 times. In the case of using MSS instead of VSS to produce sheet-metal-forming products, 1 m^2^ of MSS can save 1m^2^ of VSS which weighs 7.8 kg, so 698 kg of VSS is considered as the maximum resource conservation due to the possible area gain.

My apologies if this was not clear in the original text. The 745 kg quoted in the text does not include the weights of the engine and transmission, but only includes the weight of the virgin sheet steel which can be saved. Therefore, the analysis of the potential of the remanufacturing of WSS to MSS is only based on lightweight sheet steel. In the case of using MSS instead of VSS to produce sheet-metal-forming products, 1 m^2^ of MSS can save 1m^2^ of VSS which weighs 7.8 kg, so 745 kg of VSS is considered as the maximum resource conservation due to the possible area gain. I have carefully checked the phrasing throughout the paper to ensure that this is clear (page 10, line 5-8).
